# Effect of Age, Gender and Hearing Loss on the Degree of Discomfort Due to Tinnitus

**DOI:** 10.29252/NIRP.BCN.8.6.435

**Published:** 2017

**Authors:** Maryam Noroozian, Zahra Jafari, Elaheh Shahmiri, Shaghayegh Omidvar, Azadeh Zendehbad, Naser Amini, Mahsa Radmehr, Masoud Bagherian, Ali Yoonessi

**Affiliations:** 1. Department of Memory and Behavioral Neurology, School of Medicine, Tehran University of Medical Sciences, Tehran, Iran.; 2. Rehabilitation Research Center, Iran University of Medical Sciences, Tehran, Iran.; 3. Department of Basic Sciences, School of Rehabilitation Sciences, Iran University of Medical Sciences, Tehran, Iran.; 4. Department of Neuroscience, School of Advanced Technologies in Medicine, Tehran University of Medical Sciences, Tehran, Iran.; 5. Department of Audiology, School of Rehabilitation Sciences, Shiraz University of Medical Sciences, Shiraz, Iran.; 6. Department of Geriatric Medicine, School of Medicine, Tehran University of Medical Sciences, Tehran, Iran.; 7. Cellular and Molecular Research Center, Iran University of Medical Sciences, Tehran, Iran.; 8. Department of Audiology, School of Rehabilitation Sciences, Shahid Beheshti University of Medical Sciences, Tehran, Iran.; 9. Department of Audiology, School of Rehabilitation Sciences, Iran University of Medical Sciences, Tehran, Iran.

**Keywords:** Tinnitus, Gender, Age, Hearing loss, Discomfort

## Abstract

**Introduction:**

Tinnitus is one of the complex symptoms of hearing described as a phantom auditory sensation without any external stimulation. Due to the subjective nature of tinnitus, perception and discomfort of tinnitus vary among the patients. The main aim of this study is to investigate the effects of gender, age and the degree of hearing loss on discomfort due to tinnitus.

**Methods:**

Eighteen patients with tinnitus, aged 21–72 years, (9 males and 9 females) were recruited. Tinnitus discomfort was investigated by Tinnitus Handicap Inventory (THI) questionnaire. Psychoacoustic assessments of tinnitus and auditory threshold assessments were evaluated using a 2-channel clinical audiometer.

**Results:**

The results showed no significant correlation between THI scores with loudness matching (P=0.187), mean of auditory threshold (P=0.304), gender (P=0.93) and age (P=0.200). Also, no significant correlation was found between maximal level of hearing loss and pitch matching (P=0.208).

**Conclusion:**

The study findings suggests that tinnitus is not correlated with age, gender and hearing loss. Overall, tinnitus is a complicated clinical condition which its real impact and degree of discomfort are unclear. More investigation is needed to clarify the factors involving in tinnitus annoyance.

## 1. Introduction

Tinnitus is a phantom auditory feeling that exclusively originates from the nervous system without any corresponding mechanical or vibrating activity in the cochlea (Martines, Bentivegna et al., 2010). The highest prevalence of tinnitus is seen among 40–70 years age range group, and is almost the same for both men and women (Schleuning, 1991). Tinnitus could originate from each segment of the auditory pathway from the cochlea to the auditory cortex (Crummer & Hassan, 2004). The main obstacle in tinnitus treatment is the lack of any standard method or scale to measure reliably the intensity of tinnitus. The supplementary assessments about tinnitus include pitch matching (adaptation of tinnitus frequency with a pure voice or noise), loudness matching (estimating tinnitus intensity with a pure voice or noise), minimal level of masking (assessing the amount of voice that is needed for masking the tinnitus) and residual inhibition (decreasing or eliminating the tinnitus after exposure to a covering tone of pitch and intensity of tinnitus) (Karatas & Deniz, 2011).

In addition to the clinical evaluation, questionnaires are usually used for inspecting the individual complaints regarding tinnitus. Various types of questionnaires such as Tinnitus Handicap Questionnaire (THQ), Tinnitus Severity Questionnaire (TSQ) and Tinnitus Reaction Questionnaire (TRQ) have been used (Bahmad, Venosa, & Oliveira, 2006; Hallam, Jakes, Chambers, & Hinchcliffe, 1985; Kuk, Tyler, Russell, & Jordan, 1990). Tinnitus Handicap Inventory (THI) is one of the most varied self-assessment tools for tinnitus, which has been considerably used because of its ease of completion, functional and emotional structures, good structural validity, internal cohesion and strong test-retest reliabilities (Karatas & Deniz, 2011).

The perception of tinnitus differs in various people. It can be perceived as low, high and severe (Valente et al., 2012). Although the tinnitus annoyance and its secondary symptoms are a worldwide healthcare problem and there are numerous requests for therapeutic interventions, the impact of factors such as age, gender and hearing loss on tinnitus remains controversial to date (Seydel, Haupt, Olze, Szczepek, & Mazurek, 2013).

Gender differences regarding the tinnitus annoyance and its effect on the quality of life have been reported by many researchers (Erlandsson & Holgers, 2001; Hiller & Goebel, 2006; Lindgren, Wieslander, Dammström, & Norbäck, 2009; Meric, Gartner, Collet, & Chéry-Croze, 1998; Pinto, Sanchez, & Tomita, 2010; Seydel et al., 2013; Vanneste, Joos, & De Ridder, 2012; Welch & Dawes, 2008). Some studies have reported no relationship between gender and nuisance expected from tinnitus (Meric et al., 1998; Pinto et al., 2010; Vanneste et al., 2012), while others have confirmed this kind of relationship (Erlandsson & Holgers, 2001; Hiller & Goebel, 2006; Lindgren et al., 2009; Welch & Dawes, 2008). Moreover, considering that prevalence of hearing loss and tinnitus will increase with senility, several studies have tried to examine the relationship between tinnitus and age (Pinto et al., 2010). However, these studies have failed to reach a unique and specific conclusion. For instance, in one study (Meric et al., 1998), researchers inspected the impact of tinnitus on the quality of life, without finding any association between age and tinnitus annoyance. In another study (Hiller & Goebel, 2006), more annoyance was reported in older male patients. The impact of hearing loss on the tinnitus annoyance remains a matter of debate.

Several studies have tried to depict the possible association between amount of damage in hearing sensitivity and the scores of the THQ (Axelsson, 1991; Dias & Cordeiro, 2008; Newman, Jacobson, & Spitzer, 1996; Jayarajan, Bartlett, & Ratnayake, 2009; Savastano, 2008; Vallianatou, Christodoulou, Nestoros, & Helidonis, 2001); however, the results have been contradictory. For instance in Searchfield study, low frequency hearing loss was associated with high level of annoyance caused by tinnitus (using the THQ) (Searchfield, Jerram, Wise, & Raymond, 2007). In the mentioned study, tinnitus severity index (TSI: A questionnaire that evaluates the tinnitus annoyance) did not correlate with any audiometric findings. This poor communication suggests those patients with tinnitus are heterogeneous and that multiple factors are involved in the impact of these symptoms (Pinto et al., 2010). The relationship between hearing loss and tinnitus pitch has been inspected in several studies and different results have been reported. Some researchers observed a clear relationship between tinnitus pitch and the shape of the audiogram (Jastreboff & Hazell, 1993; Norena, Micheyl, Chéry Croze, & Collet, 2002; Roberts, Moffat, & Bosnyak, 2006; Schecklmann et al., 2012; Sereda et al., 2011), while other studies failed to report such relationship (Pan et al., 2009). Still a matter of debate, several theories about relationship between various types of hearing loss and tinnitus frequency (or pitch) have been proposed (Schecklmann et al., 2012).

Due to the subjective nature of tinnitus, the diversity of causes and the heterogeneity of patients, tinnitus is a complicated issue for study and understanding. Considering these kinds of contradictions, this study aimed to investigate the effects of gender, age and the degree of hearing loss on the annoyance caused by tinnitus. Here, the relationship between audiometric data, psychoacoustics assessments related to tinnitus and tinnitus severity were also inspected on the basis of a THI.

## 2. Methods

### 2.1. Study participants

This study was conducted on 18 patients with tinnitus aged from 21 to 72 years [mean(SD) age: 50.22(13.38) years; 9 men]. Participants were selected from patients referred to the Audiology Clinic of Tehran University of Medical Sciences. The study was conducted according to the Helsinki Declaration and its implementation was approved by the Ethics Committee of Tehran University of Medical Sciences. The patients were fully informed about the project and completed consent forms. Inclusion criteria comprised no history of exposure to noise or ototoxic drug use, lack of history of ear disease, neuro-otologic disease, psychiatric disorders, head trauma or accident, brain surgery and consumption of psychotropic drugs (Martines et al., 2010; Martines, Bentivegna, Martines, Sciacca, & Martinciglio, 2010a; 2010b). To investigate the effect of age, participants were divided into two groups of over and under 50 years old.

### 2.2. Audiometry and THI

Auditory threshold assessments and psychoacoustics evaluations of tinnitus were done using a 2-channel clinical audiometer (Madsen Orbiter 922). Auditory threshold was considered as the average pure tone for frequencies of 500, 1000, 2000, 4000 and 8000 Hz and was classified as normal hearing (<20 dB); mild hearing loss (21–40 dB); moderate hearing loss (41–70 dB); severe hearing loss (71–90 dB) and profound hearing loss (>90 dB) (Martines et al., 2010; Martines et al., 2010a; 2010b).

To evaluate the severity of tinnitus and its impact on patient’s life, THI (Mahmoudian et al., 2011) was used. THI is a 25-item questionnaire that contains three sub-scales, including functional (12 items), emotional (8 items) and catastrophic (5 items) that respectively examine physical functioning, psychological distress, despair and loss of control. Each item can be replied to with three types of answers; ‘yes’ (4 score), ‘sometimes’ (2 score) and ‘no’ (0 score), so that eventually total score ranges from 0 (no disability resulting from tinnitus) to 100 (highest tinnitus annoyance). According to these ratings, we can divide the tinnitus severity into 5 categories: slight (score 0–16), mild (score 18–36), moderate (score 38–56), severe (score 58–76) and catastrophic (score 78–100) (Mahmoudian et al., 2011).

### 2.3. Psychoacoustic assessments of tinnitus

For patients who had unilateral tinnitus, the sounds were delivered to the normal side, and for patients with central tinnitus when both ears were affected identically, the right ear was selected to deliver sounds.

### 2.4. Loudness matching

In evaluating tinnitus loudness, the patients were asked to adjust the pure-tone intensity level in 1000–16000 Hz frequency in such a way that it could be almost equal to the loudness of tinnitus (regardless of the quality of sound). At each frequency, delivering sounds began with an intensity level lower than the patient’s hearing threshold at that level of frequency, and then was increased continuously at 1 dB in steps until the person reported that loudness of external sound corresponded with the tinnitus loudness. To ensure the accuracy of assessment, the loudness matching assessments were repeated at least three times on two different days and the average score of those assessments was recorded as tinnitus loudness on a dBSL scale.

### 2.5. Pitch matching

Pitch matching was conducted using two alternate forced choice questions. Stimuli were presented in pairs and then patients were asked to identify the sound that was most consistent with the frequency of their tinnitus. Before offering each pair of sounds, the intensity level of each was set at a higher level of tinnitus loudness on the basis of the previous adaptive evaluations. Consequently, sounds delivery continued in pairs and in an intermittent manner up until the patient stated that sound frequency was closest to his or her tinnitus pitch.

### 2.6. Data analysis

Statistical analysis was performed considering the ears and not the subjects. Data that belonged to the affected side was inspected in patients with unilateral tinnitus and in patients with bilateral tinnitus both ear scores were analysed (Granjeiro et al., 2008). The Kolmogorov-Smirnov test was used to assess the normal distribution (P<0.05). Accordingly, the distributions of THI scores, the frequency with maximum hearing loss and the average auditory threshold were normal, however, it was not normal for loudness matching and pitch matching assessments. Therefore, an independent samples t-test was administered to compare THI scores between two age groups, as well as, genders. The Spearman correlation coefficient was conducted in order to inspect the correlations between THI scores and loudness matching assessments. It was also applied to determine the correlation between the frequency with the maximum hearing loss and pitch matching assessments. The Pearson correlation was used to determine the correlation between THI scores and the average auditory threshold. P values are indicated in the tables and related texts for the statistical analyses. All statistical analyses were conducted using SPSS Statistics 22 at a significance level of 0.05.

## 3. Results

The number of investigated ears was 19 (male=10); six in the right ear, 11 in the left ear, and one was perceived bilaterally. The mean and Standard Deviation (SD) of hearing thresholds was 38.71±22.78 in the investigated ears. Two ears were classified as normal hearing sensitivity, 10 with mild, three with moderate and the two with severe hearing loss (n=17 ears). The audiometric evaluation was not possible in two ears. In addition, the mean and SD of THI scores was 40.53±24.20. On the basis of THI scores, the tinnitus severity was categorized as slight in four, mild in three, moderate in seven, severe in two, and catastrophic in the two ears. The mean and SD of psychoacoustic assessments (loudness and pitch matching) according to the age and gender are shown in Table 1. This table shows that the tinnitus frequency is much lower in females than males.

**Table 1.** Mean and SD of pitch matching and loudness matching according to gender and age

		**Pitch Matching (Hz)**			**Loudness Matching (dBSL)**		

		**Mean**	**SD**	**N**	**Mean**	**SD**	**N**
Gender	Male	6000	3028.21	5	10.00	9.21	10
	Female	2250	2991.06	8	7.00	7.31	9
Age	50≥	3875	2657.54	4	11.56	9.37	10
	50<	3611.11	3913.34	9	5.90	6.51	9

SD: Standard Deviation, N: Number of ears evaluated

In addition, males perceived the tinnitus sound much louder than their female counterparts. Age did not have any effect on the frequency of tinnitus. Besides, the mean and SD of THI scores as a function of age and gender are indicated in Table 2. No significant correlation was found between THI scores with loudness matching (r=−0.317; P=0.187) and the mean of auditory threshold (r=0.265; P=0.304). Moreover, no significant correlation was found between the frequency with maximum hearing loss and pitch matching (r=0.412; P=0.208).

**Table 2.** Mean and SD of THI scores according to gender and age (Statistically significant at P<0.05)

		**THI score**			**Statistical Result**

		**Mean**	**SD**	**N**	P^*^
Gender	Male	41.00	23.84	9	0.93
	Female	40.00	26.04	9	
Age	50≥	32.89	24.15	9	0.200
	50<	47.40	23.29	9	

SD: Standard Deviation, N: Number of people evaluated

## 4. Discussion

Here, we found no difference in the annoyance caused by tinnitus between men and women. The results of the study supports the findings of those preceding studies which were unable to show any significant correlation (Erlandsson & Holgers, 2001; Fioretti, Fusetti, & Eibenstein, 2013; Meric et al., 1998; Pinto et al., 2010; Vanneste et al., 2012). However, Coelho (based on the visual analogue scale) (Coelho, Sanchez, & Bento, 2004) and Seydel (using the tinnitus questionnaire: TQ) (Seydel et al., 2013) stated that the scores of tinnitus annoyance are significantly higher in women than in men. Differences in the sample size and assessment tools make it difficult to compare the results of the aforementioned studies.

Prevalence of tinnitus and hearing loss increases with age. THI scores in patients >50 years compared to younger people, were higher but not significantly. Tinnitus is a common auditory phenomenon and more can be heard in a quiet area or by increasing the amount of auditory perception. Thus, it seems plausible that in the elderly, because of unemployment and spending more time at home, the perception of tinnitus annoyance is greater compared to younger subjects (Pinto et al., 2010). Hillerand Goebel (Hiller & Goebel, 2006) has also reported the increasing annoyance of tinnitus in older adults. This is despite the fact that two previous studies reported no effect of age on the tinnitus annoyance (Meric et al., 1998; Pinto et al., 2010).

In our study, there was also no significant correlation between the degree of hearing loss and their discomfort caused by tinnitus (based on THI score). The impact of hearing loss on the severity of annoyance caused by tinnitus remains unclear. Several studies have tried to show the possible association between THI questionnaire scores and degree of hearing loss (Axelsson, 1991; Baskill & Coles, 1999; Coles, 1984; Dias & Cordeiro, 2008; Jayarajan et al., 2009; Savastano, 2008; Vallianatou et al., 2001). A group of studies have not found any association between hearing thresholds and severity of tinnitus annoyance (Baskill & Coles, 1999; Newman et al., 1996; Vallianatou et al., 2001), others have said that people who have a higher hearing threshold gain higher THI scores (Axelsson, 1991; Coles, 1984; Newman et al., 1996; Jayarajan et al., 2009). In contrast, some researchers also said that people who have a lower hearing threshold obtain higher THI scores (Savastano, 2008). However, the results have been different and sometimes contradictory.

According to psychoacoustic assessment (loudness matching) and score THI, there was no significant correlation between THI and intensity of the tinnitus. The high intensity of the tinnitus that is perceived by patients does not mean that patients take it as a severe problem. Sweetow (Sweetow, 1986) suggests that it could be beneficial for the patients to know that there is no relation between the tinnitus loudness and its annoyance. In addition, it would be helpful for the patients to be informed about the role of other factors on the severity and tinnitus annoyance.

Here, we did not find any relationship between the frequency of the tinnitus and frequency of hearing loss on the basis of the psychoacoustics assessment (pitch matching). The findings of this study confirm some earlier studies (Figueiredo et al., 2010; Flores, Teixeira, Rosito, Seimetz, & Dall’Igna, 2015; Pan et al., 2009), but are in contrast with the results of other studies that have reported a relation between frequency of the tinnitus and frequency of hearing loss (König, Schaette, Kempter, & Gross, 2006; Martines et al., 2010; Norena et al., 2002; Schecklmann et al., 2012). By considering the fact that 90% of patients with tinnitus suffer from hearing loss and the most recent theories represent tinnitus as a central neuroplastic changes that occurs following primary cochlear injuries. The existence of a relationship between frequency of hearing loss (or adjacent frequencies) and frequency of the tinnitus is plausible. On the basis of the hypothesis of homeostatic plasticity, the central auditory system preserves neuronal homeostasis in order to maintain stability in the average of firing and neural-coding efficiency. In many cases, sensory deprivation, probably ‘neural noise’, has been reinforced due to increased gain, and this increases the neural activity in the range of deprived frequency that is eventually perceived as tinnitus (Schecklmann et al., 2012).

It seems that use of a larger sample size, classification of samples into different age groups and gender differentiated analysis can help to draw conclusions and findings into more detailed discussion. Despite the fact that THI is one of the most popular methods for evaluating the severity of tinnitus (due to easy interpretation and use and for addressing various aspects of the patient’s life) (Valente et al., 2012), using other valid tinnitus questionnaires along with THI could provide more interesting findings. Investigation of other factors such as personality, the quality of the noise in tinnitus, and duration of the symptoms may help shed a light on the variables that affect the discomfort in tinnitus.

On the basis of the findings this study, gender, age and hearing loss had no effect on the tinnitus annoyance in THI scores. Overall, the data of this study confirmed that tinnitus discomfort cannot be explained by age, gender, and hearing loss. The study of other factors in tinnitus discomfort is suggested.
